# Erratum to “Multifunctional GO Hybrid Hydrogel Scaffolds for Wound Healing”

**DOI:** 10.34133/research.0584

**Published:** 2025-02-10

**Authors:** Xiaoya Ding, Yunru Yu, Chaoyu Yang, Dan Wu, Yuanjin Zhao

**Affiliations:** ^1^ Department of Rheumatology and Immunology, Nanjing Drum Tower Hospital, School of Biological Science and Medical Engineering, Southeast University, Nanjing 210096, China.; ^2^ Oujiang Laboratory (Zhejiang Lab for Regenerative Medicine, Vision and Brain Health), Wenzhou Institute, University of Chinese Academy of Sciences, Wenzhou, Zhejiang 325001, China.

In the Research Article titled “Multifunctional GO Hybrid Hydrogel Scaffolds for Wound Healing” [1], the authors have identified an error in Figure 5. Due to an inadvertent error during the assembly of the image, panel D was incorrect. This error does not affect the results or conclusions of the paper but the authors apologize for any inconvenience this may have caused. The figure has now been corrected in the original publication and is also reproduced below.

**Figure 5.**
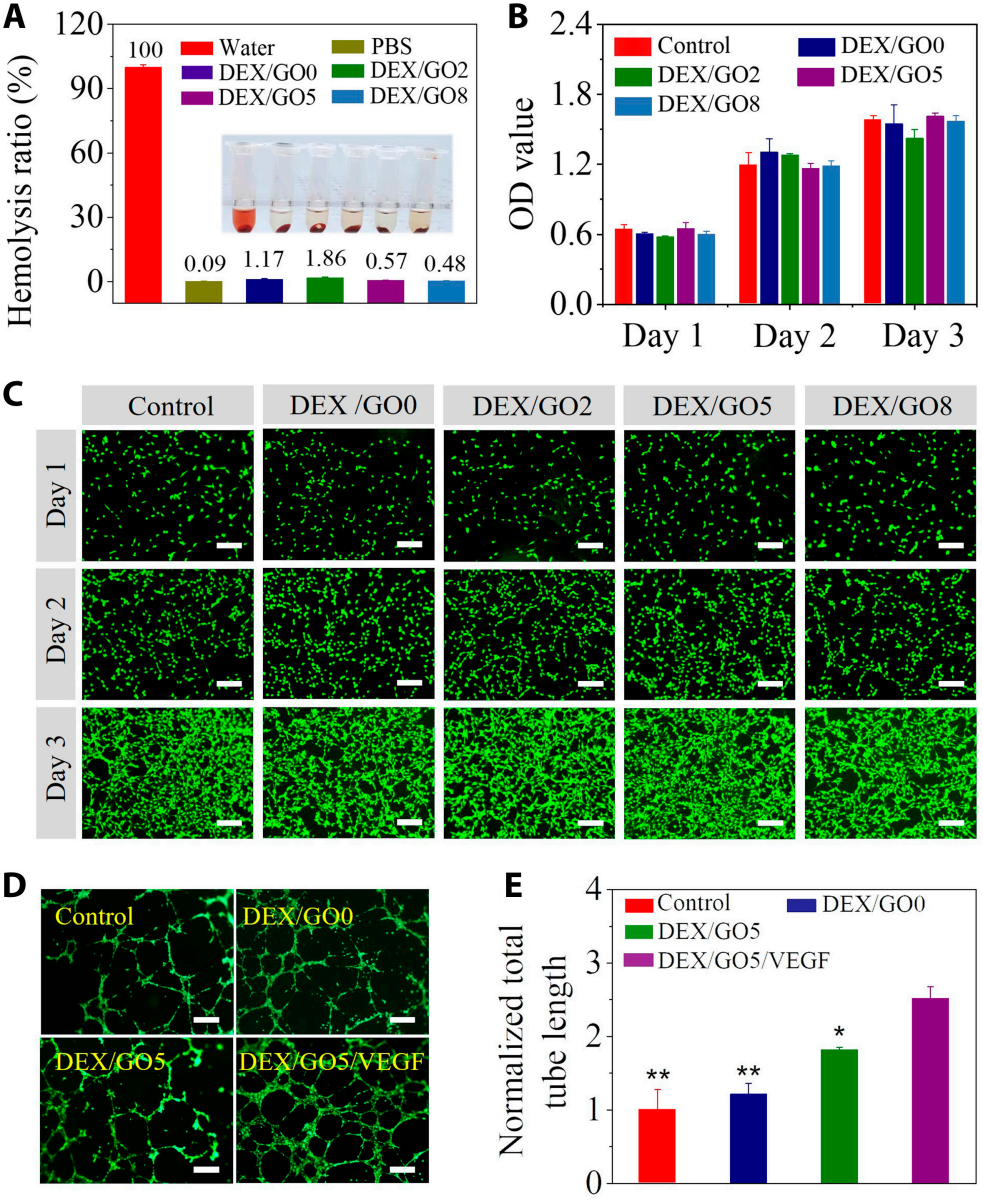
Biocompatibility of DEX/GO hydrogels. (A) The hemolytic activity of DEX/GO hydrogels with different GO contents. (B) Cytocompatibility evaluation of DEX/GO hydrogel by incubation with NIH 3T3 cells. (C) Fluorescent images of NIH 3T3 cells after contacted with DEX/GO hydrogels extract for different days. Scale bars: 200 μm. (D and E) Fluorescence images and statistical analysis of typical tubular structures of HUVECs for the VEGF-loaded DEX/GO5 hydrogels (n = 3). ∗: compared with DEX/GO5/VEGF. Scale bars: 100 μm.

## References

[1] Ding X, Yu Y, Yang C, Wu D, Zhao Y. Multifunctional GO hybrid hydrogel scaffolds for wound healing. Research. 2022;2022:9850743. 36349336 10.34133/2022/9850743 PMC9639445

