# Supplementary material for: Multifunctional GO Hybrid Hydrogel Scaffolds for Wound Healing
Source: Research (Wash D C). 2022 Oct 21;2022:9850743. doi: 10.34133/2022/9850743 (PMC9639445; doi:10.34133/2022/9850743)
Supplement: 20221021-1 [file 2022_9850743.v1.pdf]

AAAS
Research
Volume 2022, Article ID 9850743, 14 pages
https://doi.org/10.34133/2022/9850743

Research
A SCIENCE PARTNER JOURNAL

Research Article

Multifunctional GO Hybrid Hydrogel Scaffolds for Wound Healing

Xiaoya Ding,[1,2] Yunru Yu,[2] Chaoyu Yang,[2] Dan Wu,[2] and Yuanjin Zhao[1,2]

[1]*Department of Rheumatology and Immunology, Nanjing Drum Tower Hospital, School of Biological Science and Medical Engineering, Southeast University, Nanjing 210096, China*
[2]*Oujiang Laboratory (Zhejiang Lab for Regenerative Medicine, Vision and Brain Health), Wenzhou Institute, University of Chinese Academy of Sciences, Wenzhou, Zhejiang 325001, China*

Correspondence should be addressed to Yuanjin Zhao; yjzhao@seu.edu.cn

Received 19 August 2022; Accepted 29 September 2022; Published 26 October 2022

Copyright © 2022 Xiaoya Ding et al. Exclusive Licensee Science and Technology Review Publishing House. Distributed under a Creative Commons Attribution License (CC BY 4.0).

Hydrogel dressings have received extensive attention for the skin wound repair, while it is still a challenge to develop a smart hydrogel for adapting the dynamic wound healing process. Herein, we develop a novel graphene oxide (GO) hybrid hydrogel scaffold with adjustable mechanical properties, controllable drug release, and antibacterial behavior for promoting wound healing. The scaffold was prepared by injecting benzaldehyde and cyanoacetate group-functionalized dextran solution containing GO into a collection pool of histidine. As the GO possesses obvious photothermal behavior, the hybrid hydrogel scaffold exhibited an obvious stiffness decrease and effectively promoted cargo release owing to the breaking of the thermosensitive C=C double bond at a high temperature under NIR light. In addition, NIR-assisted photothermal antibacterial performance of the scaffold could be also achieved with the local temperature rising after irradiation. Therefore, it is demonstrated that the GO hybrid hydrogel scaffold with vascular endothelial growth factor (VEGF) encapsulation can achieve the adjustable mechanical properties, photothermal antibacterial, and angiogenesis during the wound healing process. These features indicated that the proposed GO hybrid hydrogel scaffold is potentially valuable for promoting wound healing and other biomedical application.

1. Introduction

Skin wounds caused by trauma or surgical operations have become a severe threat to human health and life [1–3]. Wound dressings are essential for repairing skin defects and reconstructing cutaneous functions [4–9]. Among numerous wound dressings, hydrogels exhibited significant potential for applications in wound healing since they can provide a moist environment at the wound interface, absorb the wound exudate, and facilitate the exchange of oxygen and nutrients [10–15]. Particularly, they can also act as a carrier for loading with bioactive molecules, such as anti-inflammatory drugs, bactericidal drugs, and angiogenic factors, which endow these hydrogels with the ability of anti-inflammation, antibacteria, and angiogenesis [16–18]. Thus, by applying these functional actives loaded hydrogels, wound healing could be significantly facilitated [19, 20]. However, there are some limitations of hydrogels used for wound healing. For example, soft hydrogels can support cell growth, but lack efficient spatial structure, which resulted in poor delivery of nutrients and oxygen for cells and may lead to delayed wound healing [21, 22]. In contrast, the rigid hydrogels could be facilely fabricated into fixed shapes through different technologies (e.g., microfluidics and 3D printing) [23–26], but these hydrogels were usually nondegradable, which would lead them to fail to adapt to the dynamic wound healing process [27–30]. Therefore, the development of a smart hydrogel delivery scaffold that could adapt to the dynamic wound healing process is highly anticipated.

In this study, we developed a graphene oxide (GO)-mediated photothermal responsive dextran hydrogel scaffold with adjustable mechanical properties, controllable drug release, and antibacterial behavior for facilitating wound healing, as shown in Figure 1. GO, an emerging 2D material, has attracted substantial interest owing to its fascinating properties, such as numerous active sites, high conductivity, and mechanical strength [31, 32]. Further, the additive of

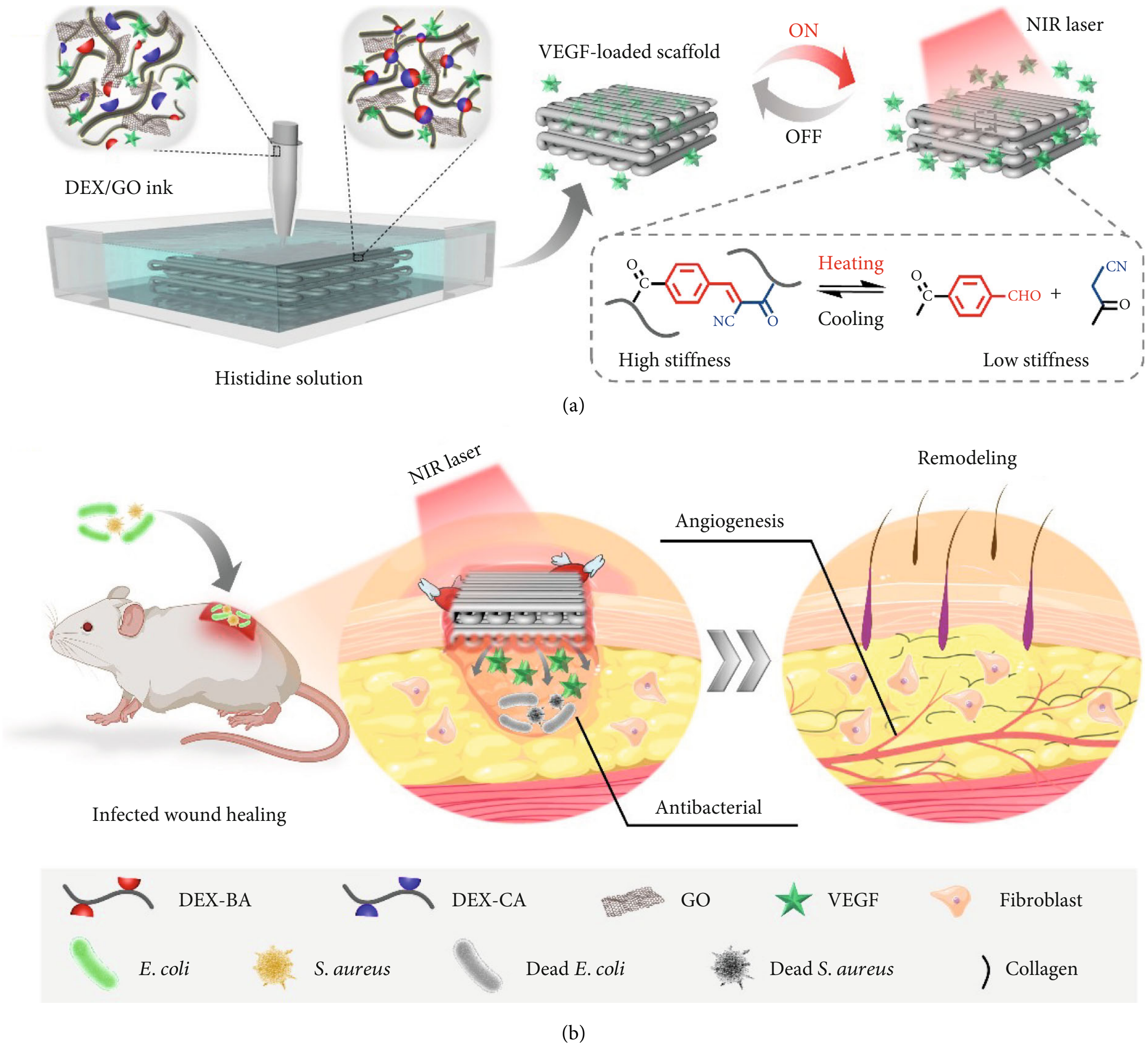

Figure 1: Schematic of the GO hybrid hydrogel scaffold fabrication through the microfluidic 3D printing technique and its application in treating infected wounds. (a) Preparation and NIR-induced stiffness change of the GO hybrid hydrogel scaffold. (b) The conductive GO hybrid hydrogel scaffold is loaded with VEGF for promoting wound healing.

GO in the hydrogel preparation could also endow these hydrogels with various unique attributes, such as high electrical conductivity, high photothermal conversion efficiency, and superior biocompatibility [33, 34]. These features make GO-loaded hydrogels valuable in biomedical fields. Besides, dextran is the neutral polysaccharide that have been widely used as starting materials for chemical modification to design new functional polymers with promising properties [35, 36]. Furthermore, it has been demonstrated that the aqueous solution of benzaldehyde and cyanoacetate groups-functionalized dextran polymers could hardly form hydrogels spontaneously in the presence of GO, but this solution could be rapidly solidified into hydrogel when the histidine was added. However, such hydrogels with adjustable mechanical properties and controllable drug release behavior remain unexplored, and the value of this hydrogel for wound healing is significantly worth being explored.

Herein, we present the desired GO hybrid hydrogels and investigated their values in wound healing. The hydrogels were printed into 3D scaffolds through a glass capillary microfluidic 3D printing technique by injecting GO-incorporated benzaldehyde and cyanoacetate group-functionalized dextran mixed solution into the histidine solutions. The resulting GO hydrogels displayed excellent photothermal performance, enhanced mechanical properties, and enhanced good electrical conductivity because of the existence of GO. In addition, the excellent photothermal performance endowed the resulting hydrogel scaffolds with adjustable mechanical properties, excellent NIR-responsive controlled drug release performance, and antibacterial. Benefiting from these features, we have demonstrated that such 3D printed GO hybrid hydrogel scaffolds loaded with vascular endothelial growth factor (VEGF) could achieve the dynamic wound healing process, photothermal antibacterial, and angiogenesis in the infected full-thickness

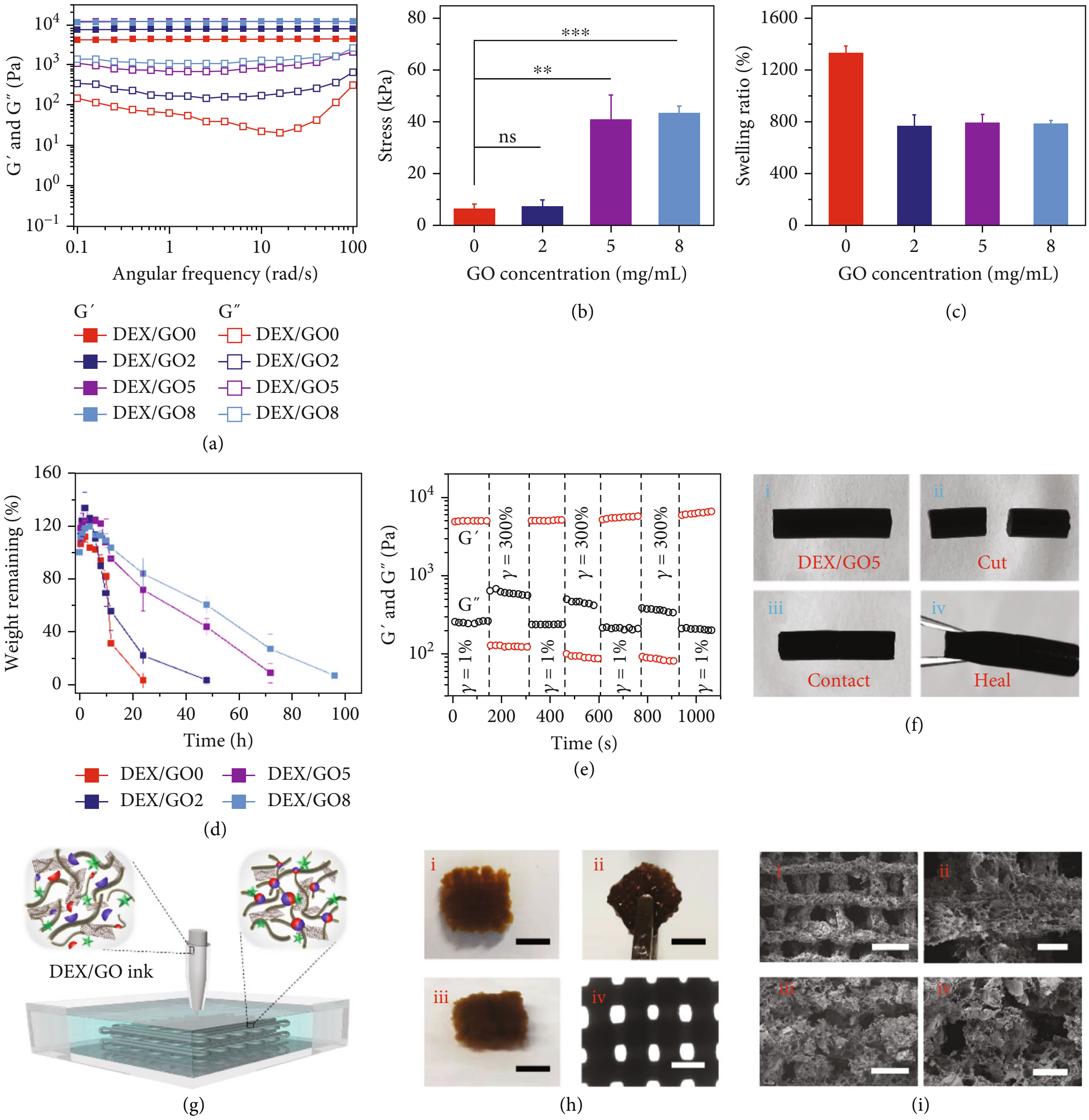

FIGURE 2: The mechanical properties, swelling, and degradation performance of the GO hybrid hydrogels and the generation of DEX/GO scaffolds through microfluidics 3D printing. (a) G' and G'' of 8% GO hybrid hydrogels with different GO concentrations as a function of the frequency from 0.1 to 100 rad/s at 37°C. (b) The compression stress of 8% GO hybrid hydrogels with different GO concentrations. ns: no significant difference. (c) Equilibrium swelling ratio of the 8% GO hybrid hydrogels after 6 h swelling. (d) Degradation of the 8% GO hybrid hydrogels with different GO concentrations at 37°C. (e) Self-healing ability of the 8% DEX/GO5 hydrogel was evaluated with an alternating strain of 1% and 300% at 37°C. (f) Images demonstrating the self-healing process for the 8% DEX/GO5 hydrogel. (g) Schematic of the microfluidics 3D printing process of GO-incorporated dextran inks (8%) in histidine solution (2%). (h) Digital images and optical microscopic images of the printed cubic scaffold. Scale bars: (i–iii) 0.5 cm and (iv) 1 mm. (i) SEM images of the printed scaffold from top (i and ii) and side (iii and iv) view. Scale bars in (i–iv) are 1.0 mm, 200 μm, 0.5 mm, and 250 μm, respectively.

skin defect model. Therefore, it is believed that the proposed 3D hydrogel scaffolds would provide a unique strategy for biomedical applications.

2. Results and Discussion

When used as wound dressings, the mechanical properties, swelling, and degradation performance of the resulting hydrogels are significantly important. In a typical experiment, the GO hybrid hydrogel could be rapidly prepared by adding histidine to the mixture solution of benzaldehyde and cyanoacetate group-functionalized dextran and GO (Figure S1). The mechanical strength for these fabricated hydrogels with varied GO concentrations was evaluated using a rheometer with a frequency ranging from 0.1 to 100 rad/s. As shown in Figure 2(a), the storage modulus (G') showed the lowest in

the hydrogel without GO (DEX/GO0 hydrogel) and increased gradually with varying GO concentrations from 2 to 8 mg/mL, indicating enhanced mechanical properties of the hydrogels. This result was further confirmed from the compression stress-strain curve. As shown in Figure 2(b) and Figure S2, the stress of the GO hybrid hydrogels was obviously increased with increasing the GO concentrations. However, upon raising the GO concentration to 8 mg/mL, the hydrogel (DEX/GO8 hydrogel) would be more brittle and may be broken at low strain (Figure S3). By immersing them in PBS (pH 7.4), these hydrogels behaved obvious swelling deformation, inferring their ability to absorb the tissue exudates when used as wound dressings (Figure 2(c)).

Apart from the equilibrated swelling ratio test, the in vitro degradation performances were also investigated. It demonstrated that the hydrogels with either the presence or the absence of GO all completely degraded within 4 days of incubation (Figure 2(d)). Specifically, the addition of GO could clearly delay the degradation process, suggesting that these hydrogel formulations have good biodegradation performance for in vivo application. Because the rapid recovery of the hydrogel strength after being destroyed is also an important feature in the practical application, the self-healing ability of the hydrogel was evaluated. The step-strain measurement displayed that the hydrogel with 5 mg/mL GO (DEX/GO5 hydrogel) was disrupted after being subjected to a high strain ($\gamma = 300\%$), while its mechanical property recovered to the original value after 1% strain was applied (Figure 2(e)). Moreover, the macroscopical observation could also be evidence of the excellent self-healing behavior of the DEX/GO5 hydrogel (Figure 2(f)). All these features demonstrated that the DEX/GO5 hydrogel was suitable for wound dressing applications.

To achieve the ability to transport oxygen, nutrients, and cells for facilitated wound healing, the hydrogel was designed and fabricated as a porous 3D scaffold via microfluidic 3D printing. Briefly, the GO mixed dextran solution was pumped through the tapered capillary and flowed into a collection bath filled with histidine solution to induce the formation of a GO hybrid hydrogel scaffold (Figure 2(g)). The printed construct exhibited superior self-standing capability and could be picked up without collapsing (Figure 2(h)). From the SEM images in Figure 2(i), we observed that the scaffold presented the well-defined microstructure as it was printed and meanwhile it maintained microporous structure, which would favor the transport of oxygen and nutrients.

Although it is not convenient to trigger the reversibility by changing temperatures when the thermal-responsive hydrogel was applied in vivo, our hydrogel was designed to be with controlled stiffness under the NIR light owing to the thermally reversible C=C double bond at higher or lower temperatures, as schemed in Figure 3(a). To verify this hypothesis, the temperature sweep test was firstly carried out for the DEX/GO5 hydrogel. As displayed in Figure 3(b) and Figure S4, G' decreased gradually when increasing the temperature from 37°C to 70°C, suggesting that the DEX/GO5 hydrogel was thermosensitive. Notably, G' of the DEX/GO5 hydrogel prepared at 37°C exhibited an obvious decrease upon heating at a fixed temperature of 45°C, 50°C, and 60°C (Figure 3(c) and Figure S5). This could be further demonstrated by larger pores of the hydrogel incubated at 50°C than 37°C for 10 min (Figure 3(c)). When the temperature was switched to 37°C again, G' and G'' showed an obvious increase owing to the rebuilding of the dynamic C=C double bond (Figure 3(d) and Figure S6). These results confirmed that the GO hybrid hydrogel exhibited obvious thermally reversible properties, and the addition of GO has no effect on the thermal reversibility of the hydrogel based on the C=C double bond.

Despite that it will not affect thermal reversibility, the existence of GO endowed the hybrid hydrogel with photothermal characteristics. To study the photothermal performance of the DEX/GO hydrogels, an 808 nm laser (1 W/cm^2) was applied on these GO hybrid hydrogels for 5 min, and then was absent for 3 min. Thermal infrared images of the heating process showed the excellent photothermal properties of these DEX/GO hydrogels (Figure 3(e)). In detail, the real-time temperatures were recorded. It could be seen that the temperatures of these hydrogels increased rapidly and reached a stable state of 29.7°C, 44°C, 53.5°C, and 57.2°C within 5 min, respectively (Figure 3(f)). Besides, the photothermal performance of the DEX/GO5 hydrogel could be controlled under changed laser power densities (Figure S7). The excellent photothermal stability of the DEX/GO5 hydrogel could also be proved by cycled irradiation and cooling processes. As shown in Figure 3(g), four cycles of irradiation temperature rise were almost identical from 27.7°C to 50°C. Taking together, all these results confirmed the excellent temperature-responsive and photothermal performance of the GO hybrid hydrogel.

The thermo-induced stiffness changes of GO hybrid hydrogels using NIR facilitated the controllable drug release (Figure 4(a)). To demonstrate this, rhodamine B was firstly loaded into the precursor during the GO hybrid hydrogel formation. Figure 4(c) displayed the bioactive molecules release from the hydrogel during ON/OFF NIR irradiation cycles. In detail, irradiation of the GO hybrid hydrogel stimulated a fast drug release, which was in accordance with a low-stiffness state under irradiation. While switching off the light showed a relatively slower release, which should be attributed to the high stiffness without irradiation. As a comparison, the drug release from this hydrogel without irradiation was used as a control, and it displayed a slower release from the hydrogel. Similarly, a model protein FITC-BSA was incorporated into the GO hybrid hydrogel as well. As shown in Figure 4(d), similar switchable drug release behavior from the GO hybrid hydrogel could be also demonstrated. Thus, it could be concluded that the thermo-induced stiffness changes for GO hybrid hydrogels stimulated the controlled drug release, and this cyclic ON/OFF irradiation onto the hydrogel offers a promising strategy to control the released dose of the loaded drugs.

Studies have demonstrated that conductive hydrogels could obviously facilitate wound healing process. [37–40] Hence, the conductivity of the GO hybrid hydrogels was

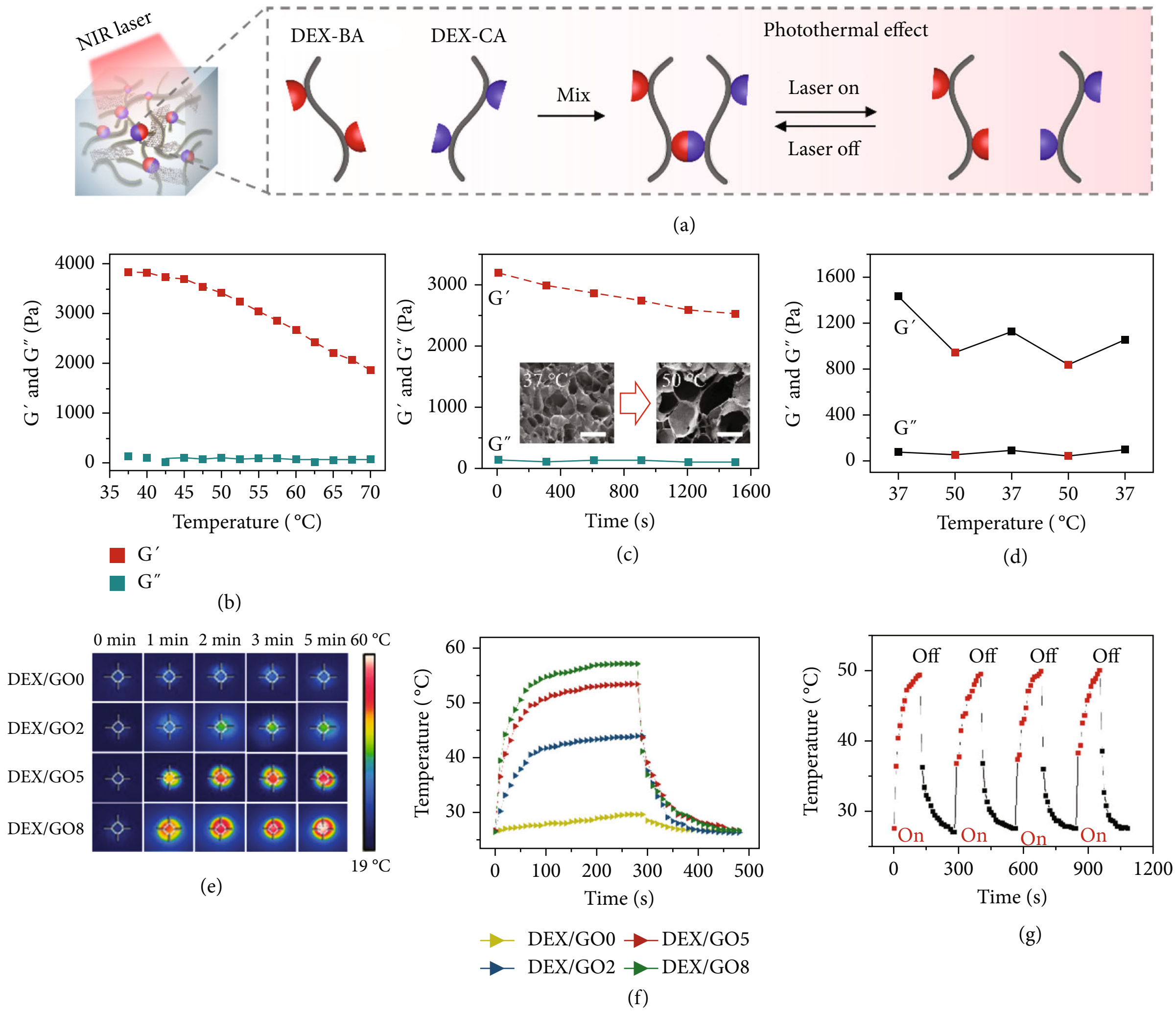

Figure 3: Thermo-responsive performance of DEX/GO hydrogels. (a) NIR-induced thermo-responsive stiffness control of the 8% DEX/GO hybrid hydrogel. (b) Variation in G' and G'' of the 8% DEX/GO5 hydrogel with temperatures. (c) G' changes of 8% DEX/GO5 hydrogel incubated for 25 min at 50°C. SEM images of the 8% DEX/GO5 hydrogels formed at 37°C and incubated at 50°C for 10 min (insert). Scale bars: 150 μm. (d) Switchable stiffness control of the DEX/GO5 hydrogel at 37°C and 50°C. (e and f) Thermo-responsive cycles of the hybrid hydrogel with different GO contents and the corresponding real-time infrared thermal imaging pictures. (g) The real-time temperature of DEX/GO hydrogel under cycled photoirradiation.

carried out. It was shown that the conductivity of the hydrogel without GO was the lowest and the increasing concentration of GO would elevate its conductivity (Figure 4(e)). Moreover, the conductivity of the DEX/GO5 hydrogel would also increase when the temperature of the hydrogel was raised, which may attribute to the fact that the lower stiffness of the hydrogel provides more opportunities for GO in the hydrogel to connect at high temperatures (Figure 4(f)). This conductivity enabled the DEX/GO5 hydrogel to transfer the electricity from a 3 V supply to light the LED lamp (Figures 4(b) and 4(g)) . Although the physical rupture of the hydrogel would cause the lighting-off, it would be lighted again when the separated pieces were brought into contact and self-healed. The reason may be that the dynamic C=C double bond crosslinking as well as the conduction paths at the interface reformed again when the separated hydrogels were put together. This result could be further proved in Figure 4(h). These results demonstrated that the DEX/GO hydrogels had excellent conductive and self-healing abilities.

Good biocompatibility is indispensable when the material was contact with tissues. Thus, blood compatibility of the DEX/GO hydrogels was first evaluated. As shown in Figure 5(a), there is no difference among the hydrogel groups and PBS group, but obvious hemolysis behavior was observed for the water group. The quantitative results displayed that hemolysis ratio of hydrogel groups was insufficient to result in hematotoxicity for the tissue, suggesting good blood compatibility of these DEX/GO hydrogels. Apart from this, the cell biocompatibility of these hydrogels was also carried out via CCK-8 assay, and it displayed that the viability of NIH 3T3 cells treated with the DEX/GO hydrogel

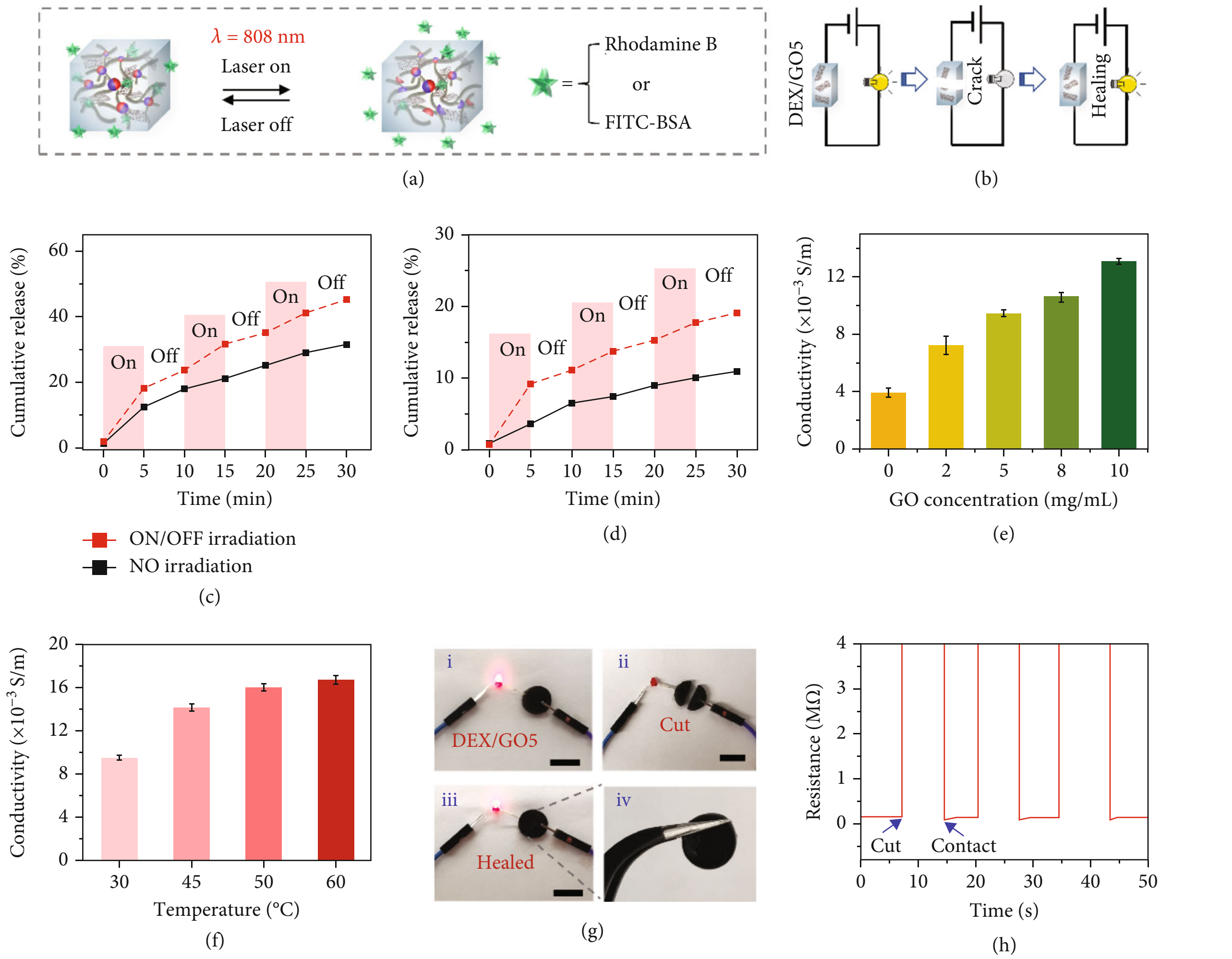

Figure 4: NIR-responsive drug release and conductivity ability of the DEX/GO hydrogels. (a) The scheme demonstrating the NIR-responsive controlled drugs delivery. (b) The schematic showing the electrical self-healing ability of 8% DEX/GO5 hydrogel. (c) Switchable release of rhodamine B from the 8% DEX/GO5 hydrogels upon switching on and off the NIR laser light. (d) Controllable FITC-BSA release from 8% DEX/GO5 hydrogels upon switching on and off NIR laser. (e) The conductivity of the 8% hydrogels with different GO contents. (f) The conductivity of the 8% DEX/GO5 hydrogels at different temperatures. (g) Optical images demonstrating the good conductivity of the 8% DEX/GO5 hydrogel during a breaking/healing cycle. Scale bars: (i–iii) 1 cm. (h) Electrical self-healing ability of the 8% DEX/GO5 hydrogel showing the resistance variation during the cut/healing process.

extracts maintained about 100% after 24, 48, or 72 h of incubation, indicating that the DEX/GO hydrogels were noncytotoxic (Figure 5(b)). Live/dead cell viability assay was further performed to estimate the cytocompatibility of DEX/GO hydrogels (Figure 5(c)). It was found that almost no dead cells were observed after treatment with the DEX/GO hydrogel extracts, and normal cell proliferation occurred. In addition to good biocompatibility, vascular endothelial growth factors (VEGF) that could promote blood vessel formation were encapsulated into the GO hybrid hydrogel to facilitate the wound healing process. In order to verify whether bioactivity of the released VEGF from the DEX/GO hydrogels under NIR light was maintained, the tube formation assay was carried out by using human umbilical vein endothelial cells (HUVECs). As shown in Figure 5(d), compared to the HUVECs cultured in the cell medium (control group), HUVECs cultured in DEX/GO5 hydrogel extracts containing VEGF have developed more tubular structures. Figure 5(e) further demonstrated that the total tube length for the DEX/GO5 hydrogel loaded with VEGF was higher than that in other groups. To conclude, the DEX/GO5 hydrogel had good biocompatibility, and the released VEGF from the DEX/GO5 hydrogel under NIR light could effectively induce the blood vessel formation.

Studies have shown that the temperature of 50°C displayed negligible damage to the wound tissue but high efficiency on antimicrobial infection. [41, 42] Thus, based on the good photothermal performance of the GO hybrid hydrogels, the NIR-assisted photothermal antibacterial ability was evaluated against *E. coli*, *S. aureus*, and *C. albicans* in vitro by directly irradiating the samples with NIR light

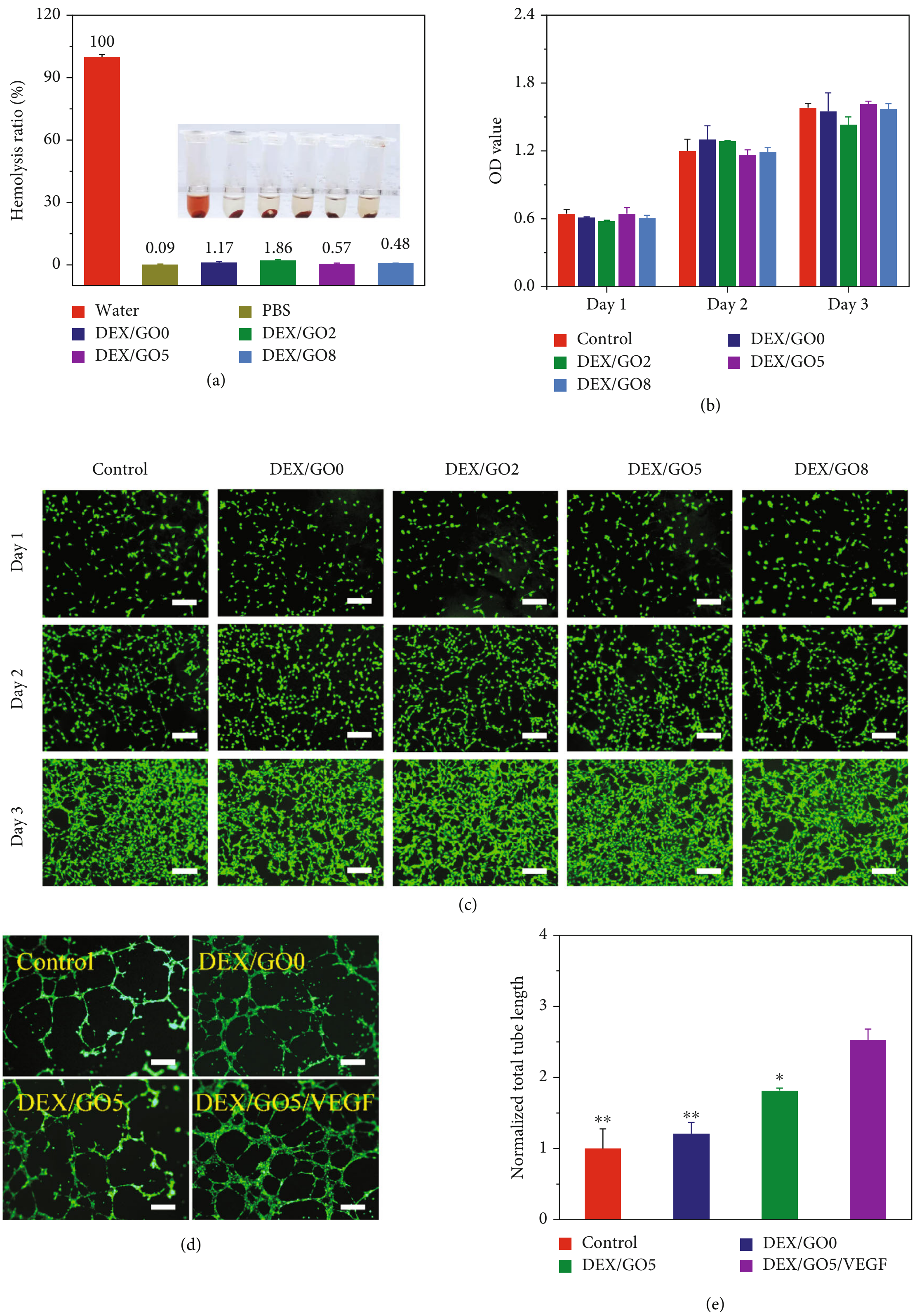

FIGURE 5: Biocompatibility of DEX/GO hydrogels. (a) The hemolytic activity of DEX/GO hydrogels with different GO contents. (b) Cytocompatibility evaluation of DEX/GO hydrogel by incubation with NIH 3T3 cells. (c) Fluorescent images of NIH 3T3 cells after contacted with DEX/GO hydrogels extract for different days. Scale bars: 200 μm. (d and e) Fluorescence images and statistical analysis of typical tubular structures of HUVECs for the VEGF-loaded DEX/GO5 hydrogels ($n = 3$). *: compared with DEX/GO5/VEGF. Scale bars: 100 μm.

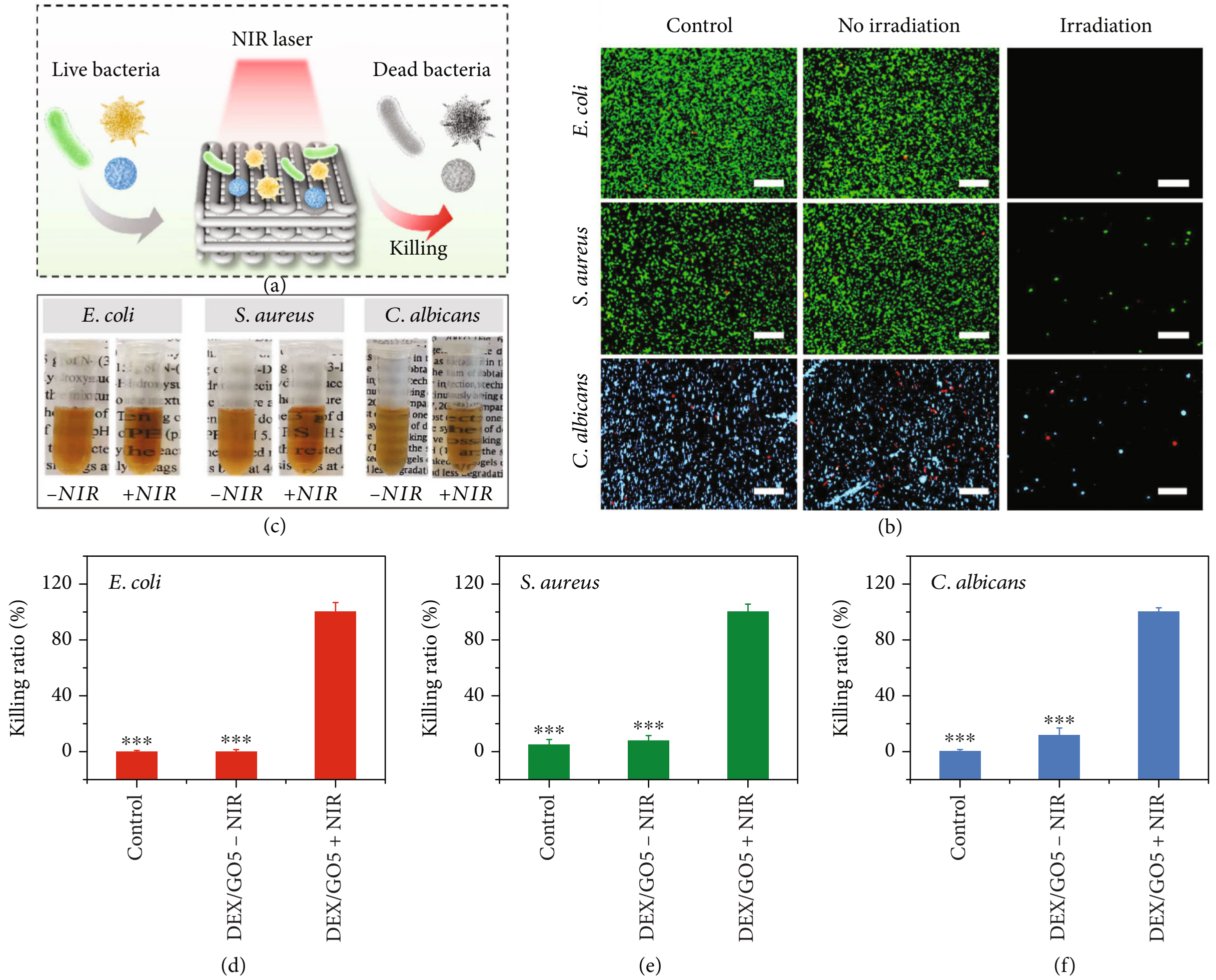

Figure 6: The photothermal antibacterial property of DEX/GO hydrogels. (a) Schematic diagram of the antibacterial principle of DEX/GO hydrogels. (b) Live/dead bacterial staining of *E. coli*, *S. aureus*, and *C. albicans* incubated with DEX/GO5 hydrogels with or without NIR irradiation. Scale bars: 100 μm. (c) The images of the bacteria incubated with DEX/GO5 hydrogels for 24 h with or without NIR irradiation. (d–f) The quantitative bacterial killing ratio of DEX/GO5 hydrogels with or without NIR irradiation. *: compared with DEX/GO5+NIR.

(Figure 6(a)). It could be found that almost no live bacteria were observed when DEX/GO5 hydrogel was exposed to the NIR irradiation for 5 min. As a comparison, most of the bacteria were alive in the absence of NIR irradiation, which is similar to the control group (Figure 6(b)). Also, these culture mediums were photographed, as shown in Figure 6(c). For the hydrogels cultured with the bacterial with NIR irradiation, the culture mediums were very clear. In contrast, the culture mediums were turbid owing to the growth of the residual bacterial. Furthermore, the quantitative bacterial killing ratio of DEX/GO5 hydrogels with the presence or absence of NIR irradiation was evaluated. As displayed in Figures 6(d)–6(f), when NIR laser was applied to DEX/GO5 hydrogel for 5 min, the bacteria-killing ratio against *E. coli*, *S. aureus*, and *C. albicans* was all about 100%. As a control, the bacterial killing ratio of the DEX/GO5 hydrogels without NIR irradiation was negligibly different when compared to the control group. Taken together, DEX/GO5 hydrogels with NIR irradiation exhibited outstanding antibacterial performance.

Benefiting from the abovementioned features of GO hybrid hydrogel, the wound healing ability of the DEX/GO5 hybrid hydrogel scaffolds loaded with VEGF was evaluated by establishing full-thickness skin wounds infected by *S. aureus*. The rats were treated differently, including those with PBS, DEX hydrogel without GO (DEX/GO0 group), DEX/GO5 hydrogel scaffold (DEX/GO5 group), DEX/GO5 hydrogel scaffold loaded with VEGF but not received NIR irradiation (DEX/GO5/VEGF-NIR group), and VEGF-loaded DEX/GO5 hydrogel scaffold irradiated with 808 nm for 5 min (DEX/GO5/VEGF+NIR group), respectively. The healing results were recorded and photographed on day 0, 2, 5, 7, and 11 before the dressing change (Figure 7(a)). It was obvious that the DEX/GO5 group exhibited a better recovery compared to the DEX/GO0 group after treatment for 2 days because the conductive hydrogel was beneficial to wound healing. However, the wound healing of the DEX/GO5 group was delayed owing to the bacterial infection and no statistically significant change was seen between these two treatment groups after being treated for 11 days. In

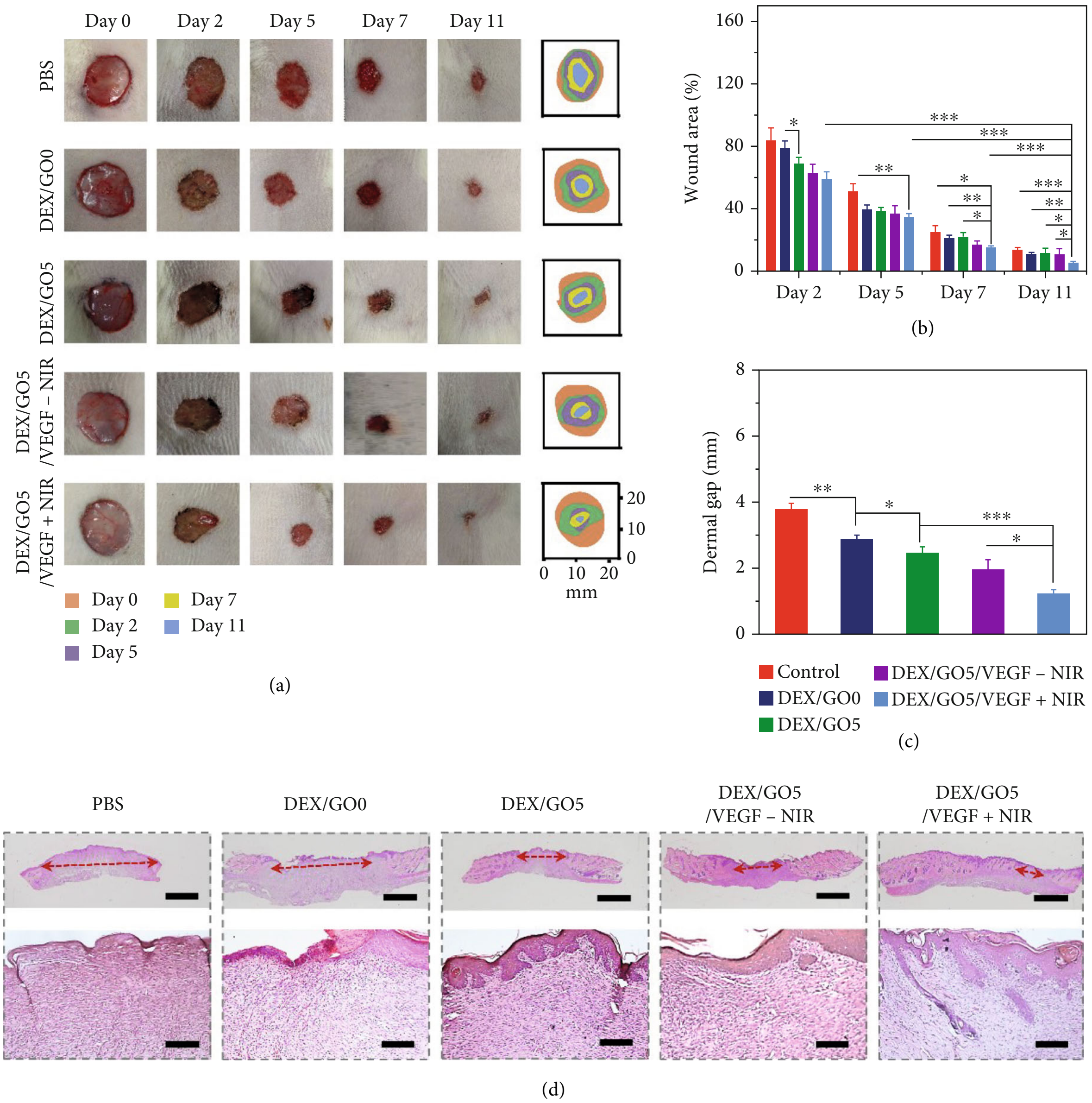

FIGURE 7: Wound healing performance of the hydrogel in a full-thickness skin defect model. (a) Wounds images in rats after being treated with PBS, DEX/GO hydrogel (DEX/GO0), DEX/GO5 hydrogel scaffold (DEX/GO5), DEX/GO5 hydrogel scaffold loaded with VEGF but without NIR irradiation (DEX/GO5/VEGF-NIR), and DEX/GO5 hydrogel scaffold loaded with VEGF and meanwhile irradiated using NIR for 5 min (1 W/cm^2) (DEX/GO5/VEGF+NIR). (b) Quantitative analysis of wound areas of each group ($n = 6$). (c) The quantitative width of wound edges of each group. (d) H&E staining of wound tissues after being treated for 11 days. The below columns are the magnified image of the corresponding top column (The red arrows indicated microscopic wound edges). Scale bars are 400 μm (top) and 100 μm (below), respectively.

addition, it presented an obvious closure in the DEX/GO5/VEGF-NIR group compared with the DEX/GO5 group owing to the promoting angiogenesis performance of the loaded VEGF. Particularly, for DEX/GO5/VEGF+NIR group, a significantly accelerated wound closure rate compared to other groups was observed after treatment for 11 days, and the wounds were almost healed and covered with hair. This may be attributed to the photothermal antibacterial and controlled drug delivery performance of the GO hybrid hydrogel under NIR irradiation.

According to the calculated wound closure area at different time points, the wound area treated with DEX/GO5/VEGF+NIR group (5.3% ± 0.8%) was smaller than that of the DEX/GO5 group (11.1% ± 3.5%) and DEX/GO5/VEGF-NIR group (10.5% ± 3.6%) after treatment for 11 days (Figure 7(b)). In addition, from the hematoxylin-eosin (H&E) staining results in Figures 7(c) and 7(d), the dermis layer and skin appendages (e.g., hair follicle) were regenerated at the wound site in the DEX/GO5/VEGF+NIR group. Furthermore, the average length of wound edge was 1249 ± 109 μm in the DEX/GO5/VEGF+NIR group and 1952 ± 313 μm in the DEX/GO5/VEGF-NIR group, which was greatly smaller than that in the other groups. Besides, the DEX/GO5/VEGF+NIR group had the maximum width of

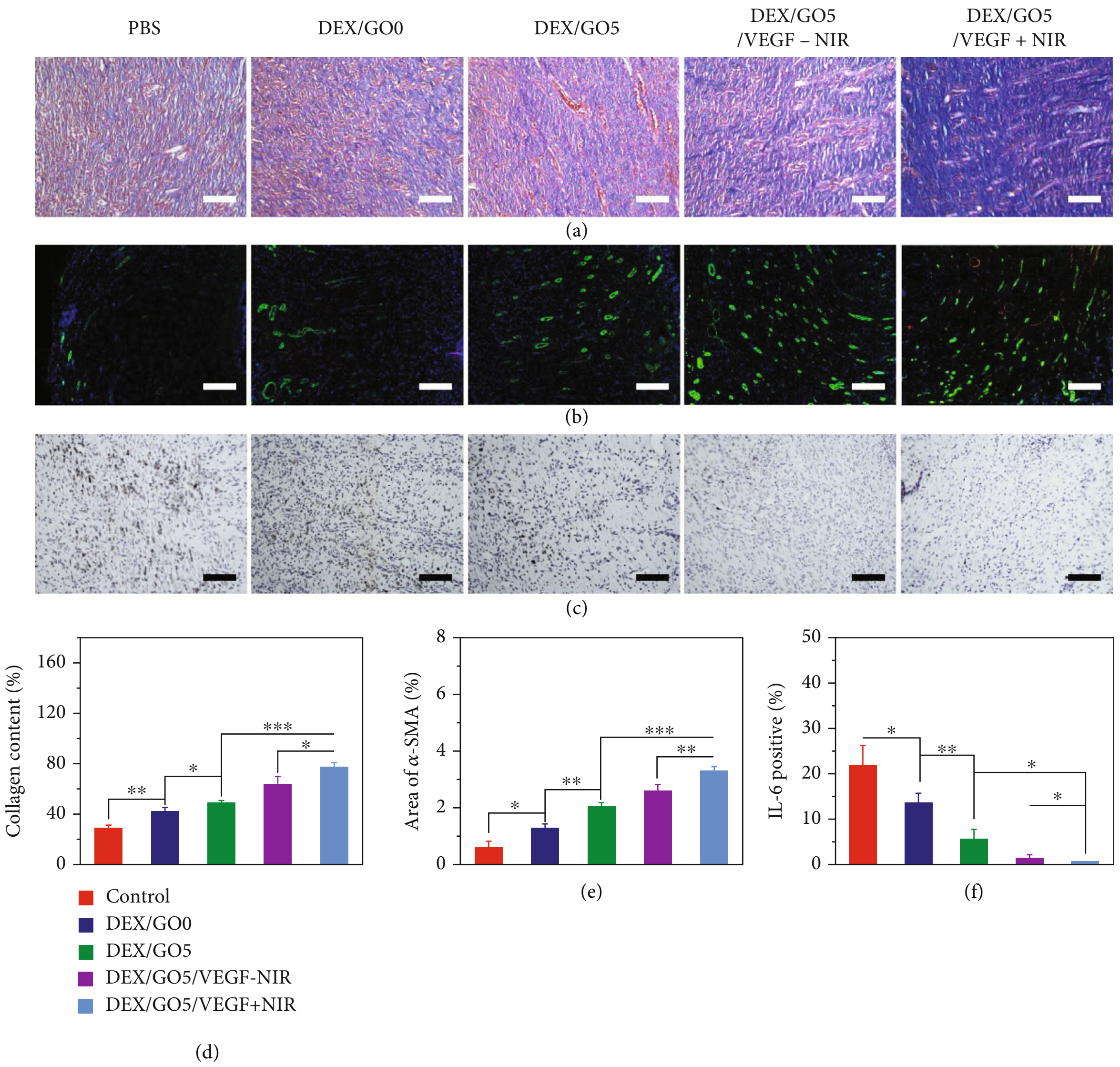

Figure 8: Accelerated collagen deposition, promoted angiogenesis, and reduced inflammation. (a) Collagen deposition content after 11 days of wound healing. Scale bars: 100 μm. (b) Immunofluorescence assay of CD31 and α-SMA to indicating vascularization. Scale bars: 200 μm. (c) The immunohistochemistry of IL-6 of the granulation tissues for the different groups. Scale bars: 100 μm. (d) The collagen content (%) in the different groups calculated from Figure 7(a). (e) Statistical analysis of the relative coverage area of the α-SMA. (f) Statistical analysis of the IL-6 contents in the different groups.

epidermis thickness among all these treated groups (Figure S8).

The wound healing process was further evaluated by collagen deposition, angiogenesis, and inflammation. Masson's trichrome staining was firstly performed to detect collagen deposition. In detail, the DEX/GO5/VEGF+NIR group displayed denser and well-organized collagen fibers in the wound bed, while for the other groups, there was less amount of collagen formed (Figures 8(a) and 8(d)). This result indicated that the DEX/GO5/VEGF+NIR group exhibited improved tissue remolding performance. To indicate the neovascularization, double immunofluorescence of CD31 (vascular endothelial cell marker indicating new vessels) and α-SMA (α-smooth muscle actin, vascular smooth muscle cells, and mature blood vessels) were carried out. It demonstrated that the PBS group exhibited lower CD31 and α-SMA expression, which suggested that few vascular structures were formed at the wound site (Figure 8(b)). This result was probably because there was excess aggregation of inflammatory cells at the wound bed caused by the bacteria. In contrast, the rest of groups showed a higher expression ratio of CD31 and α-SMA, especially in DEX/GO5/VEGF+NIR group, which could be ascribed to the promoted vascularization of the released VEGF and the effective photothermal antibacterial capability of the DEX/GO5 hydrogel scaffold under NIR irradiation (Figure 8(e)). Furthermore, the immunohistochemical staining for a proinflammatory cytokine of interleukin-6 (IL-6) in the regenerated tissues was used to evaluate the inflammatory response, which can reflect the infection level of the wounds caused by the

bacteria during the healing process. As shown in Figures 8(c) and 8(f), the PBS group expressed a high level of IL-6, suggesting a serious inflammatory response. However, the lowest expression of IL-6 was detected in DEX/GO5/VEGF +NIR group. Therefore, these results demonstrated that the DEX/GO5/VEGF+NIR treated group was conducive to tissue remolding, vascularization, and anti-inflammatory and further presented excellent wound healing performance.

3. Conclusions

In conclusion, we have presented a novel conductive graphene oxide (GO) hybrid hydrogel scaffold with adjustable mechanical properties, controllable drug release, and antibacterial behavior for promoting infected wound healing. The GO hybrid hydrogel scaffold exhibited an obvious stiffness decrease, effectively promoting cargo release and photothermal antibacterial performance under NIR light. When loaded with VEGF and served as dressings to treat the infected full-thickness wounds, the scaffold could thus achieve adjustable mechanical properties, photothermal antibacterial, and angiogenesis during the wound healing process. These features demonstrated that the proposed NIR-responsive GO hybrid hydrogel scaffold would provide insights into the development of multifunctional hydrogel scaffold dressings for precision treatment in infected wound healing and other biomedical applications.

4. Materials and Methods

4.1. Materials and Cells. Dextran (Mn = 70,000 g mol^{-1}) was purchased from Aladdin. GO suspension (20 mg/mL) was obtained from XF NANO Materials Tech Co. (Nanjing China). 4-Formylbenzoic acid and cyanoacetic acid were bought from Alfa Aesar. Histidine was bought from Aladdin. 4-Dimethylaminopyridine (DMAP) was bought from Energy Chemical. 1-Ethyl-3-(3-dimethylaminopropyl) carbodiimide (EDC·HCl) was bought from GL Biochem Ltd. Dimethyl sulfoxide (DMSO) was obtained from Aladdin. Cell Counting Kit-8, calcein-AM, and propidium iodide were obtained from Sigma-Aldrich. Bovine albumin (BSA) and cell culture media were bought from Gibco. NIH 3T3 cells were purchased from the American Type Culture Collection (Rockville, MD) and were grown in Dulbecco's modified Eagle's medium (DMEM).

4.2. Characterizations. The morphologies of hydrogels and scaffolds were obtained from a scanning electron microscope (Hitachi SU8010, Japan). Optical microscopic photographs of scaffolds could be obtained on a stereomicroscope (Olympus BX51, Japan). Flow rate of the polymer solutions was controlled by the syringe pump (LSP01-2A, Halma plc, England). The rheology characterization was carried out on a rotational rheometer (DHR-2, USA). The compression test was evaluated by a mechanical testing machine (5944, Instron, USA). The resistance for these hydrogels was monitored by a digital multimeter (DMM6500, Keithley, Beaverton, USA).

4.3. Synthesis of Benzaldehyde and Cyanoacetate End-Functionalized Dextran. The benzaldehyde and cyanoacetate modified dextran were prepared by a simple chemical conjugation reaction using EDC·HCl and DMAP as the coupling reagents. Typically, dextran (5.0 g) was firstly added into DMSO in a flask and then 4-formylbenzoic acid (0.93 g, 0.25 eq. to dextran dimer unit), EDC·HCl (5.0 g, 1 eq.), and DMAP (0.11 g, 0.03 eq.) were added into dextran solution. After reaction for 2 days, the solution was purified by dialyzing against water and then lyophilized to give the final product (Yield: ~91%). Similarly, the cyanoacetate modified dextran, of which the substitution degree of the cyanoacetate groups was about 100%, was prepared by using the same method as described above (Yield: ~94%).

4.4. Preparation and Characterization of GO Hybrid Hydrogels. DEX-CA and DEX-BA were dissolved in 150 μL GO suspensions (molar ratio cyanoacetate: benzaldehyde = 1 : 1; GO concentrations: 0, 2, 5, and 8 mg/mL). Subsequently, the histidine (2%) was added to the GO mixed dextran solutions and the resulting solution could transit from sol to gel immediately. The concentration of the GO hybrid hydrogel was expressed as a weight (DEX-CA and DEX-BA)/volume percentage (w/v, %).

The mechanical characterization of GO hybrid hydrogels was evaluated on a DHR-2 rotational rheometer with 8 mm diameter steel parallel-plate geometry. The frequency sweep tests of the GO hybrid hydrogels with different GO concentrations were performed with frequencies ranging from 0.1 Hz to 100 Hz. Then, to confirm the temperature responsiveness of the GO hybrid hydrogels, a temperature sweep test of the hydrogel with a GO concentration of 5 mg/mL was firstly carried out with a temperature ranging from 37°C to 70°C. In addition, the storage modulus changes of the GO hybrid hydrogel formed at 37°C were also recorded as a function of time at temperatures of 45°C, 50°C, and 60°C, respectively. Furthermore, self-healing property of the GO hybrid hydrogel was carried out using continuous step-strain measurements. For this measurement, the GO hybrid hydrogel was formed at 37°C for 600 s, and whereafter, a strain of 300% and 1% were alternately applied onto the hydrogel to break and restore the network, respectively. This process was repeated four times.

4.5. Fabrication of the GO Hybrid Hydrogel Scaffold. The GO hybrid hydrogel scaffold was prepared by using a capillary microfluidic device. The capillaries with diameters of 300 μm were immobilized on glass slides with needles and epoxy resin. GO mixed dextran (DEX/GO) solution that was comprised of 8% DEX-BA and DEX-CA mixed solution and GO (5 mg/mL) was prepared. The DEX/GO5 solution was filled in a syringe and then injected into the microfluidic device through polyethylene tubes. A syringe pump was used to control the flow rate (2 mL/h) of the DEX/GO solutions and the GO hybrid hydrogel scaffold could be formed in a histidine collection bath.

4.6. The Degradation of the GO Hybrid Hydrogels. The GO hybrid hydrogels were prepared by adding the histidine to 500 μL DEX/GO solutions with different GO concentrations. The vial (W_p) containing GO hydrogel was accurately weighed (W_0). Then, the obtained hydrogels were immersed in 1 mL PBS solution (pH 7.4) and then incubated at 37°C.

At predetermined time intervals, PBS media were changed using new PBS, and the remaining hydrogel was weighed (W_t). The percentage of remaining hydrogel mass was calculated from the equation: $SR = (W_t - W_0)/(W_0 - W_p)$. All of the experiment was performed in triplicate.

4.7. Drug Release Test of the GO Hybrid Hydrogels. To encapsulate bioactive molecules into the GO hybrid hydrogels, FITC-BSA (1 mg/mL) was firstly mixed with 0.5 mL GO mixed hydrogel precursor solutions. Then, the histidine (10 mg/mL) was added into the precursor solutions to fabricate the FITC-BSA-loaded GO hybrid hydrogels. The FITC-BSA loaded GO hybrid hydrogels in tubes with 2 mL of PBS (pH 7.4) were incubated at 37°C with shaking speed of 100 rpm. In order to investigate the NIR triggered FITC-BSA release from the GO hybrid hydrogels, the hydrogel (0.5 mL) with 2 mL of PBS were irradiated with the NIR light (1 W/cm^2) for 5 min. The FITC-BSA-loaded GO hybrid hydrogels without receiving NIR light were used as a control. At the predetermined time intervals, 0.5 mL PBS was taken out from the tube and 0.5 mL fresh PBS was added into the tube. The concentrations of FITC-BSA were measured by UV-vis-NIR spectrophotometer. Similarly, the release of rhodamine B (1 mg/mL) was tested by using the same method described above.

4.8. Photothermal Performance of GO Hybrid Hydrogels. Photothermal performance of GO hybrid hydrogels with different GO concentrations (0, 2, 5, 8 mg/mL) was evaluated under the 808 nm laser. Briefly, the GO hybrid hydrogels (300 μL) were placed on the marble. The real-time temperature and the thermal photos of these hydrogels were measured on an infrared thermal imaging camera and recorded for 5 min with the 808 nm laser on, and then turn off the laser to cool for 2 min. This process was repeated 5 times. Moreover, the photothermal performance of DEX/GO5 hydrogel under different light intensities (0.25, 0.5, 0.75, and 1 W/cm^2) was also evaluated according to the above process.

4.9. The Hemolysis Assays. The erythrocytes were separated by centrifugation (1000 rpm). Then, they were washed using PBS for three times and diluted to 5% (v/v). GO hybrid hydrogels (500 μL) with different GO concentrations and erythrocytes solutions (500 μL) in were placed into tubes, and subsequently they were shaken in the incubator at 37°C for 120 min. Next, the erythrocyte solutions in the tubes were centrifuged and the supernatant (100 μL) was transferred to a 96-well microplate. The absorbance was detected at 576 nm on a microplate reader. PBS buffer was chosen as the negative control and the water as the positive control. The hemolysis percentage could be determined based on

$$\text{Hemolysis}\,(\%) = \left[\frac{A_g - A_p}{A_w - A_p}\right] \times 100\% \quad (1)$$

where A_g, A_w, and A_p represent the absorbance of hydrogel groups, water groups, and PBS groups, respectively.

4.10. The Cytotoxicity of GO Hybrid Hydrogels. Biocompatibility of GO hybrid hydrogels were tested by using CCK-8 assay and Live/dead cell staining. Before each test, GO hydrogels were first irradiated with UV light overnight, and then it was immersed in a serum-containing medium and incubated for 24 hours. For the cell cytotoxicity analysis, NIH 3T3 cells (10^3/well) were cultured in DMEM for 12 h. And then the medium was changed using 200 μL filtrate of GO hydrogels. CCK-8 assay was carried out after incubation for 24, 48, and 72 hours. Similarly, for the Live/dead cell assay, at the predetermined times, fluorescence images were recorded via a fluorescence microscope.

4.11. The Antibacterial Property of GO Hybrid Hydrogels. The GO hybrid hydrogel cubes (2 cm × 2 cm) were prepared and then placed into the 6-well plate, and bacterial suspension (10 μL, 10^8 CFU/mL) was added onto the surface of these hydrogels. Then, NIR laser (808 nm, 1.0 W/cm^2) was applied onto the GO hybrid hydrogel for 2 min. Subsequently, 5 mL of the sterilized culture medium was applied to resuspend all the bacteria and incubated at 37°C overnight. Finally, 100 μL of the above bacterial resuspension was transferred into 96-well and OD value at 610 nm was measured.

4.12. The Use of GO Hybrid Hydrogel Scaffold for Wound Healing In Vivo. The female Sprague Dawley (SD) rats (200-250 g) were bought from Beijing Vital River Laboratory Animal Technology Co., Ltd. All animal experiments were carried out on the basis of the Laboratory Animal Care and Use Guidelines and approved by the Animal Care and Use Committee of Wenzhou Medical University (Zhejiang, China). SD rats were anesthetized and then the back hair was removed. Wounds (diameter: 1.5 cm) were created onto the back and then 100 μL of *S. aureus* suspensions (10^8 CFU/mL) were dripped onto the wound surface to establish the infection wound models. These infection wounds were treated with PBS solution, DEX/GO0 hydrogel, DEX/GO5 hydrogel scaffold, VEGF-loaded DEX/GO5 hydrogel scaffold without NIR irradiation (DEX/GO5/VEGF-NIR), and VEGF-loaded DEX/GO5 hydrogel scaffold with NIR irradiation (DEX/GO5/VEGF+NIR), respectively ($n = 6$). In order to prevent evaporation, these wounds were covered with commercially film dressings to keep moist environment. Photographs of wounds were taken on days 0, 2, 5, 7, and 11, and the size of these wound was determined from this equation: wound size $(\%) = (A_t/A_0) \times 100\%$, in which A_t and A_0 represents the wound size on day t and day 0, respectively.

In order to further evaluate the wound healing effect, the skin around the wound site were harvested on day 11. After being fixed in 4% paraformaldehyde for 2 days, these tissues were embedded in paraffin and sectioned into 5 μm thick slices. Afterwards, H&E, Masson's trichrome staining, immunofluorescence, and immunohistochemistry were carried out and tissue slices were photo-captured using a microscope (ZEISS Axio Vert.A1).

4.13. Statistical Analyses. All results in the experiment were shown as mean ± SD. The data were evaluated by Student's t-test and the difference was considered as $^*p < 0.05$, $^{**}p < 0.01$, and $^{***}p < 0.001$.

Data Availability

All data needed to evaluate the conclusions in the paper are present in the paper and/or the Supplementary Materials. Additional data related to this paper may be requested from the authors.

Conflicts of Interest

The authors declare that there is no conflict of interest regarding the publication of this article.

Authors' Contributions

Y.J.Z. conceived the idea and designed the experiment; X.Y.D. carried out the experiments and analyzed the data. Y.R.Y., C.Y.Y., and D.W. contributed to scientific discussion of the article.

Acknowledgments

This work was supported by the National Key Research and Development Program of China (2020YFA0908200), the National Natural Science Foundation of China (52073060 and 61927805), and the Shenzhen Fundamental Research Program (JCYJ20190813152616459 and JCYJ2021 0324133214038).

Supplementary Materials

Figure S1: the digital images of DEX hydrogel and the DEX/GO hydrogel formation process. Figure S2: the stress-strain curve of the DEX/GO hydrogels with different GO concentrations. Figure S3: the strain sweep test of the DEX/GO hydrogels with different GO concentrations. Figure S4: the G' and G'' of DEX/GO0 hydrogel at 50°C at different time. Figure S5: the G' and G'' of DEX/GO5 hydrogels at 45°C and 60°C at different time. Figure S6: the G' and G'' of DEX/GO5 hydrogel at 37°C and 50°C, respectively. Figure S7: the thermal infrared images and photothermal heating curves of the DEX/GO5 hydrogels at different laser power intensities. Figure S8: the quantitative width of epidermis thickness for all groups. *(Supplementary Materials)*

References

[1] C. H. Lim, Q. Sun, K. Ratti et al., "Hedgehog stimulates hair follicle neogenesis by creating inductive dermis during murine skin wound healing," *Nature Communications*, vol. 9, no. 1, pp. 1–13, 2018.

[2] P. Rousselle, F. Braye, and G. Dayan, "Re-epithelialization of adult skin wounds: cellular mechanisms and therapeutic strategies," *Advanced Drug Delivery Reviews*, vol. 146, pp. 344–365, 2019.

[3] C. K. Sen, "Human wounds and its burden: an updated compendium of estimates," *Advanced Wound Care*, vol. 8, no. 2, pp. 39–48, 2019.

[4] K. S. Bryan, S. Zurab, and A. K. Paul, "Advances in skin grafting and treatment of cutaneous wounds," *Science*, vol. 346, no. 6212, pp. 941–945, 2014.

[5] B. Guo, R. Dong, Y. Liang, and M. Li, "Haemostatic materials for wound healing applications," *Nature Reviews Chemistry*, vol. 5, no. 11, pp. 773–791, 2021.

[6] G. C. Gurtner, S. Werner, Y. Barrandon, and M. T. Longaker, "Wound repair and regeneration," *Nature*, vol. 453, no. 7193, pp. 314–321, 2008.

[7] V. Parfejevs, J. Debbache, O. Shakhova et al., "Injury-activated glial cells promote wound healing of the adult skin in mice," *Nature Communications*, vol. 9, no. 1, p. 236, 2018.

[8] N. G. Ricapito, C. Ghobril, H. Zhang, M. W. Grinstaff, and D. Putnam, "Synthetic biomaterials from metabolically derived synthons," *Chemical Reviews*, vol. 116, no. 4, pp. 2664–2704, 2016.

[9] D. Simões, S. P. Miguel, M. P. Ribeiro, P. Coutinho, A. G. Mendonça, and I. J. Correia, "Recent advances on antimicrobial wound dressing: a review," *European Journal of Pharmaceutics and Biopharmaceutics*, vol. 127, pp. 130–141, 2018.

[10] G. Chen, Y. Yu, X. Wu, G. Wang, J. Ren, and Y. Zhao, "Bioinspired multifunctional hybrid hydrogel promotes wound healing," *Advanced Functional Materials*, vol. 28, no. 33, p. 1801386, 2018.

[11] R. Dimatteo, N. J. Darling, and T. Segura, "*In situ* forming injectable hydrogels for drug delivery and wound repair," *Advanced Drug Delivery Reviews*, vol. 127, pp. 167–184, 2018.

[12] C. Ghobril and M. W. Grinstaff, "The chemistry and engineering of polymeric hydrogel adhesives for wound closure: a tutorial," *Chemical Society Reviews*, vol. 44, no. 7, pp. 1820–1835, 2015.

[13] Z. R. Jia, J. L. Gong, Y. Zeng et al., "Bioinspired conductive silk microfiber integrated bioelectronic for diagnosis and wound healing in diabetes," *Advanced Functional Materials*, vol. 31, no. 19, p. 2010461, 2021.

[14] Y. Liang, J. He, and B. Guo, "Functional hydrogels as wound dressing to enhance wound healing," *ACS Nano*, vol. 15, no. 8, pp. 12687–12722, 2021.

[15] J. Ouyang, Q. Bu, N. Tao et al., "A facile and general method for synthesis of antibiotic-free protein-based hydrogel: wound dressing for the eradication of drug-resistant bacteria and biofilms," *Bioactive Materials*, vol. 18, pp. 446–458, 2022.

[16] D. Liu, H. Zhang, F. Fontana, J. T. Hirvonen, and H. A. Santos, "Microfluidic-assisted fabrication of carriers for controlled drug delivery," *Lab on a Chip*, vol. 17, no. 11, pp. 1856–1883, 2017.

[17] L. Siebert, E. Luna-Cerón, L. E. García-Rivera et al., "Light-controlled growth factors release on tetrapodal ZnO-incorporated 3D-printed hydrogels for developing smart wound scaffold," *Advanced Functional Materials*, vol. 31, no. 22, p. 2007555, 2021.

[18] J. Yang, H. Zhang, T. Hu et al., "Recent advances of microneedles used towards stimuli-responsive drug delivery, disease theranostics, and bioinspired applications," *Chemical Engineering Journal*, vol. 426, p. 130561, 2021.

[19] X. Zhao, Y. Liu, C. Shao et al., "Photoresponsive delivery microcarriers for tissue defects repair," *Advanced Science*, vol. 6, no. 20, p. 1901280, 2019.

[20] Y. Zhuang and W. Cui, "Biomaterial-based delivery of nucleic acids for tissue regeneration," *Advanced Drug Delivery Reviews*, vol. 176, p. 113885, 2021.

[21] Q. Feng, J. Xu, K. Zhang et al., "Dynamic and cell-infiltratable hydrogels as injectable carrier of therapeutic cells and drugs for treating challenging bone defects," *ACS Central Science*, vol. 5, no. 3, pp. 440–450, 2019.

[22] Y. Tan, H. Huang, D. C. Ayers, and J. Song, "Modulating viscoelasticity, stiffness, and degradation of synthetic cellular niches via stoichiometric tuning of covalent versus dynamic noncovalent cross-linking," *ACS Central Science*, vol. 4, no. 8, pp. 971–981, 2018.

[23] N. D. Dolinski, Z. A. Page, E. B. Callaway et al., "Solution mask liquid lithography (SMaLL) for one-step, multimaterial 3D printing," *Advanced Materials*, vol. 30, no. 31, p. 1800364, 2018.

[24] S. H. Kim, Y. K. Yeon, J. M. Lee et al., "Precisely printable and biocompatible silk fibroin bioink for digital light processing 3D printing," *Nature Communications*, vol. 9, no. 1, pp. 1–14, 2018.

[25] Y. Yu, L. Shang, J. Guo, J. Wang, and Y. Zhao, "Design of capillary microfluidics for spinning cell-laden microfibers," *Nature Protocols*, vol. 13, no. 11, pp. 2557–2579, 2018.

[26] H. Zhang, G. Chen, Y. Yu, J. Guo, Q. Tan, and Y. Zhao, "Microfluidic printing of slippery textiles for medical drainage around wounds," *Advanced Science*, vol. 7, no. 16, p. 2000789, 2020.

[27] Y. Dong, M. Rodrigues, X. Li et al., "Injectable and tunable gelatin hydrogels enhance stem cell retention and improve cutaneous wound healing," *Advanced Functional Materials*, vol. 27, no. 24, p. 1606619, 2017.

[28] Y. Li, X. Wang, Y. Fu, Y. Wei, L. Zhao, and L. Tao, "Self-adapting hydrogel to improve the therapeutic effect in wound-healing," *ACS Applied Materials & Interfaces*, vol. 10, no. 31, pp. 26046–26055, 2018.

[29] X. Mao, R. Cheng, H. Zhang et al., "Self-healing and injectable hydrogel for matching skin flap regeneration," *Advanced Science*, vol. 6, no. 3, p. 1801555, 2019.

[30] X. Sun, Q. Lang, H. Zhang et al., "Electrospun photocrosslinkable hydrogel fibrous scaffolds for rapid in vivo vascularized skin flap regeneration," *Advanced Functional Materials*, vol. 27, no. 2, p. 1604617, 2017.

[31] C. Korupalli, H. Li, N. Nguyen et al., "Conductive materials for healing wounds: their incorporation in electroactive wound dressings, characterization, and perspectives," *Advanced Healthcare Materials*, vol. 10, no. 6, p. 2001384, 2021.

[32] X. Yu, H. Cheng, M. Zhang, Y. Zhao, L. Qu, and G. Shi, "Graphene-based smart materials," *Nature Reviews Materials*, vol. 2, no. 9, pp. 1–13, 2017.

[33] B. Zhang, J. He, M. Shi, Y. Liang, and B. Guo, "Injectable self-healing supramolecular hydrogels with conductivity and photo- thermal antibacterial activity to enhance complete skin regeneration," *Chemical Engineering Journal*, vol. 400, p. 125994, 2020.

[34] X. Zhao, B. Guo, H. Wu, Y. Liang, and P. X. Ma, "Injectable antibacterial conductive nanocomposite cryogels with rapid shape recovery for noncompressible hemorrhage and wound healing," *Nature Communications*, vol. 9, no. 1, pp. 1–17, 2018.

[35] I. Cumpstey, "Chemical modification of polysaccharides," *International Scholarly Research Notices*, vol. 2013, Article ID 417672, 27 pages, 2013.

[36] G. Sun, X. Zhang, Y. Shen et al., "Dextran hydrogel scaffolds enhance angiogenic responses and promote complete skin regeneration during burn wound healing," *Proceedings of the National Academy of Sciences*, vol. 108, no. 52, pp. 20976–20981, 2011.

[37] L. C. Kloth, "Electrical stimulation technologies for wound healing," *Advances in Wound Care*, vol. 3, no. 2, pp. 81–90, 2014.

[38] Y. Wu, L. Wang, B. Guo, Y. Shao, and P. X. Ma, "Electroactive biodegradable polyurethane significantly enhanced Schwann cells myelin gene expression and neurotrophin secretion for peripheral nerve tissue engineering," *Biomaterials*, vol. 87, pp. 18–31, 2016.

[39] Y. Zhao, Z. Li, S. Song et al., "Skin-inspired antibacterial conductive hydrogels for epidermal sensors and diabetic foot wound dressings," *Advanced Functional Materials*, vol. 29, no. 31, p. 1901474, 2019.

[40] C. W. Hsiao, H. L. Chen, Z. X. Liao et al., "Effective photothermal killing of pathogenic bacteria by using spatially tunable colloidal gels with nano-localized heating sources," *Advanced Functional Materials*, vol. 25, no. 5, pp. 721–728, 2015.

[41] M. C. Wu, A. R. Deokar, J. H. Liao, P. Y. Shih, and Y. C. Ling, "Graphene-based photothermal agent for rapid and effective killing of bacteria," *ACS Nano*, vol. 7, no. 2, pp. 1281–1290, 2013.

[42] S. M. Yu, G. W. Li, P. H. Zhao et al., "NIR-laser-controlled hydrogen-releasing PdH nanohydride for synergistic hydrogen-photothermal antibacterial and wound-healing therapies," *Advanced Functional Materials*, vol. 29, no. 50, p. 1905697, 2019.
