# Supplementary material for: Multifunctional GO Hybrid Hydrogel Scaffolds for Wound Healing
Source: Research (Wash D C). 2022 Oct 21;2022:9850743. doi: 10.34133/2022/9850743 (PMC9639445; doi:10.34133/2022/9850743)
Supplement: Supplementary Materials — Figure S1: the digital images of DEX hydrogel and the DEX/GO hydrogel formation process. Figure S2: the stress-strain curve of the DEX/GO hydrogels with different GO concentrations. Figure S3: the strain sweep test of the DEX/GO hydrogels with different GO concentrations. Figure S4: the G′ and G″ of DEX/GO0 hydrogel at 50°C at different time. Figure S5: the G′ and G″ of DEX/GO5 hydrogels at 45°C and 60°C at different time. Figure S6: the G′ and G″ of DEX/GO5 hydrogel at 37°C and 50°C, respectively. Figure S7: the thermal infrared images and photothermal heating curves of the DEX/GO5 hydrogels at different laser power intensities. Figure S8: the quantitative width of epidermis thickness for all groups. [file 9850743.f1.docx]

**Supporting Information**

**Multifunctional GO hybrid hydrogel scaffolds for wound healing**

Xiaoya Ding ^1,2^, Yunru Yu ^2^, Chaoyu Yang ^2^, Dan Wu ^2^, Yuanjin Zhao ^1,2,*^

*^1^ Department of Rheumatology and Immunology, Nanjing Drum Tower Hospital, School of Biological Science and Medical Engineering, Southeast University, Nanjing 210096, China*

*^2^ Oujiang Laboratory (Zhejiang Lab for Regenerative Medicine, Vision and Brain Health), Wenzhou Institute, University of Chinese Academy of Sciences, Wenzhou, Zhejiang 325001, China*

^*^Corresponding author: Prof. Yuanjin Zhao, Southeast University, Nanjing, 210096, China. E-mail: yjzhao@seu.edu.cn


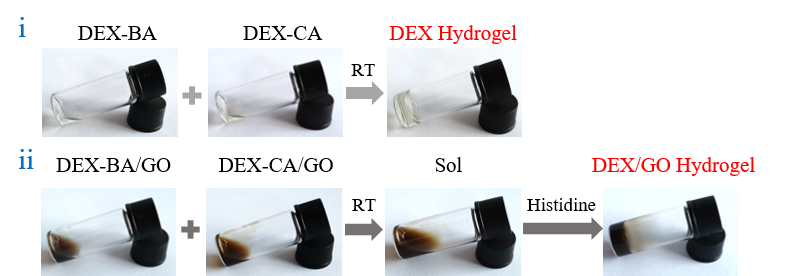


**Figure S1**. The digital images of DEX hydrogel and the DEX/GO hydrogel formation process.


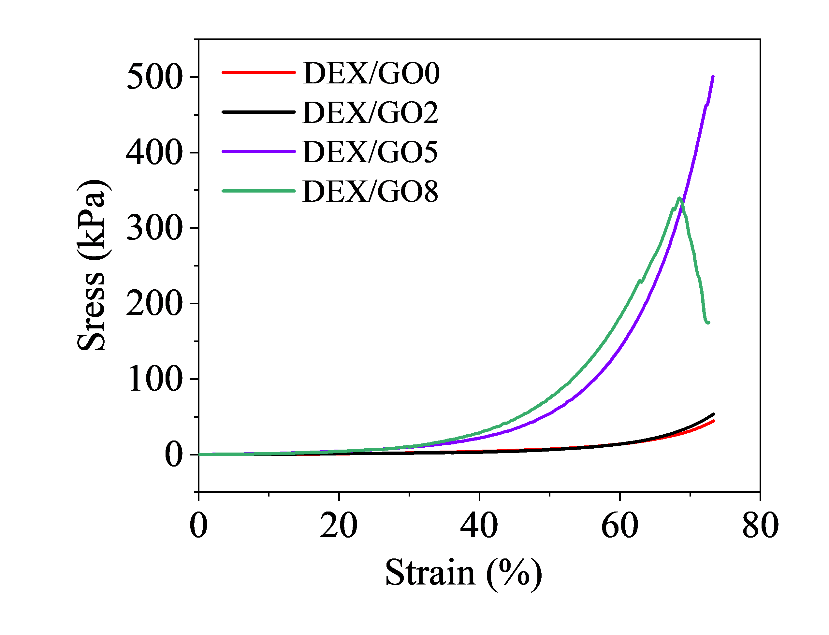


**Figure S2**. The stress-strain curve of the DEX/GO hydrogels with different GO concentrations.


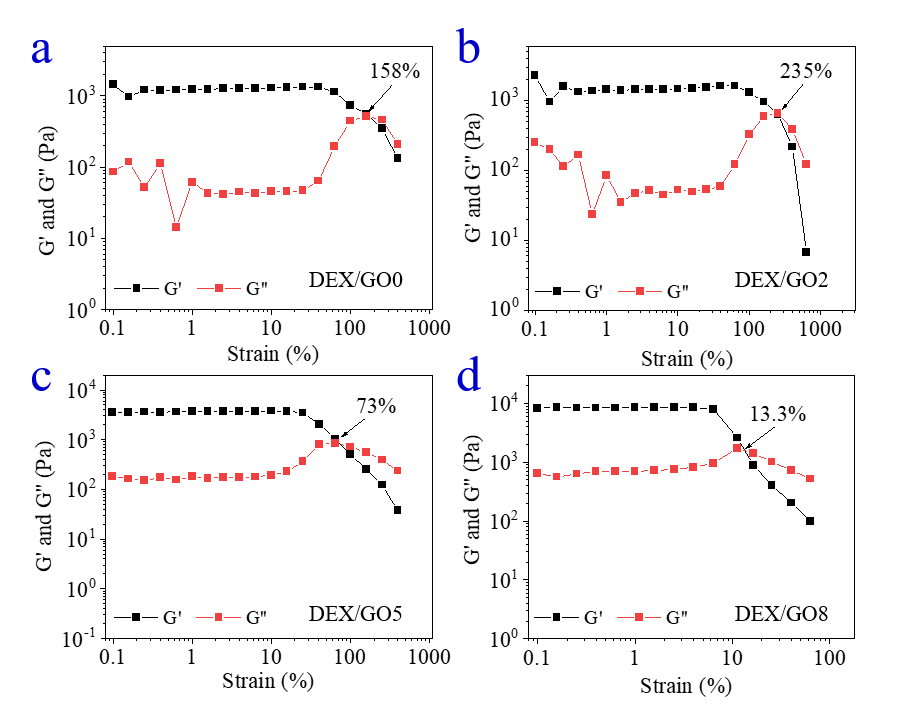


**Figure S3**. The strain sweep test of the DEX/GO hydrogels with different GO concentrations.


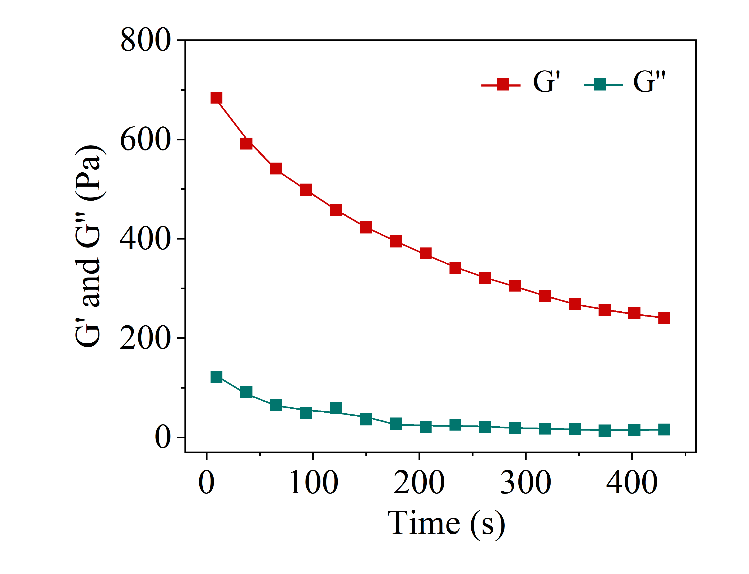


**Figure S4**. The *G'* and *G''* of DEX/GO0 hydrogel at 50 ℃ at different time.


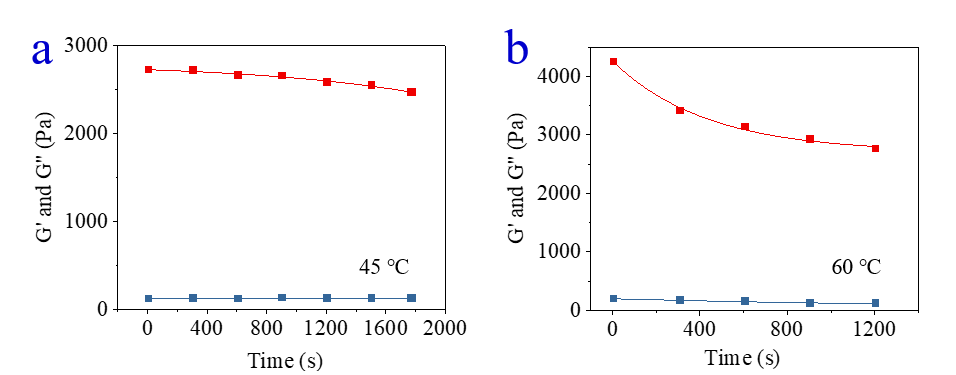


**Figure S5**. The *G'* and *G''* of DEX/GO5 hydrogels at a) 45 ℃ and b) 60 ℃ at different time.


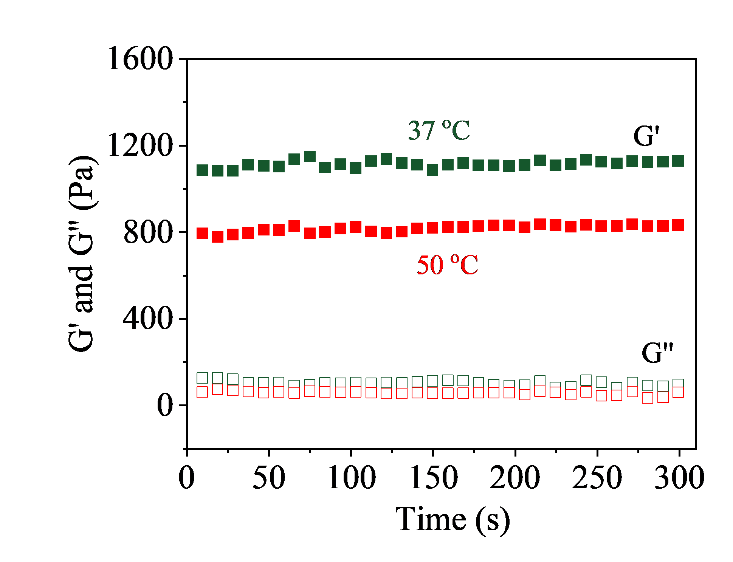


**Figure S6**. The *G'* and *G''* of DEX/GO5 hydrogel at 37 ℃ and 50 ℃, respectively.


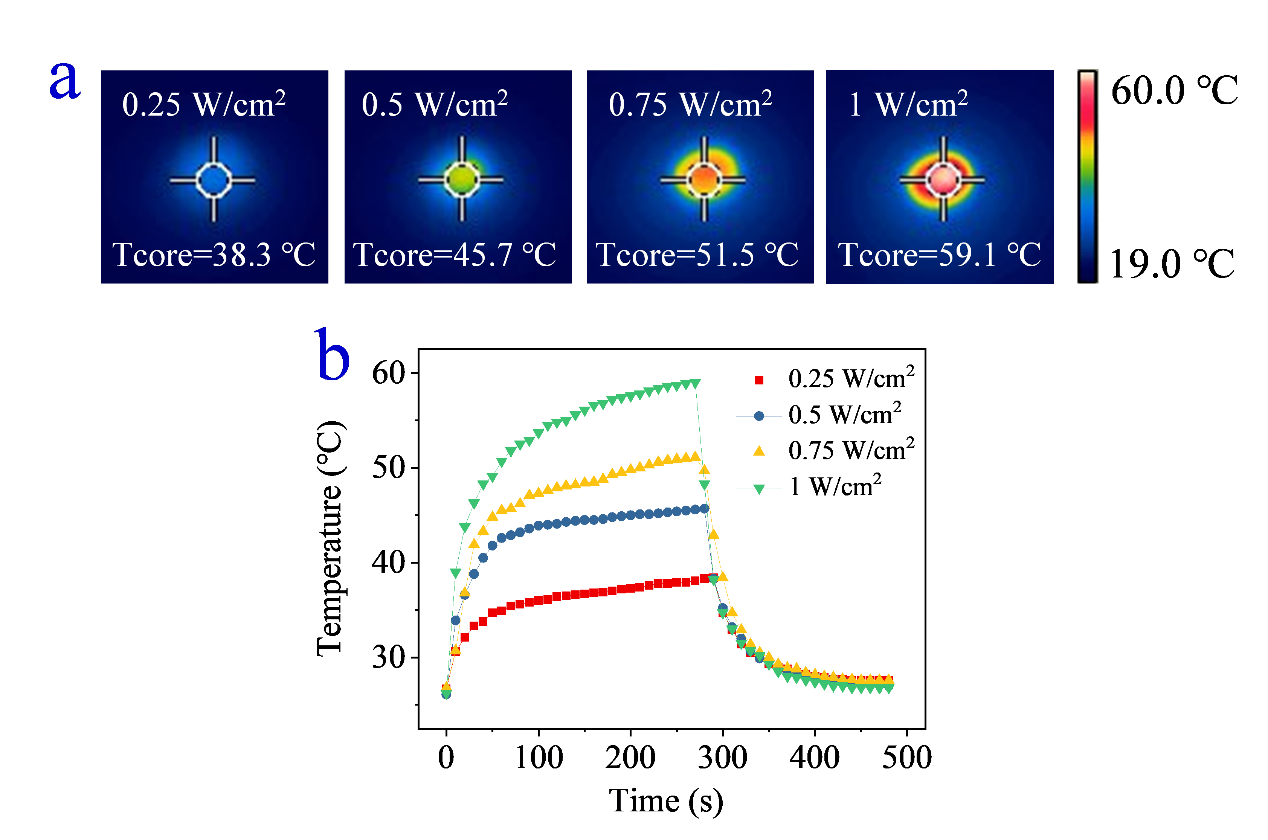


**Figure S7**. a) The thermal infrared images of the DEX/GO5 hydrogels under different laser power intensities (0.25, 0.5, 0.75, and 1 W/cm^2^). b) The photothermal heating curves of the DEX/GO5 hydrogels at different laser power intensities (0.25, 0.5, 0.75, and 1 W/cm^2^) for 300 s and afterwards cooling for 120 s.


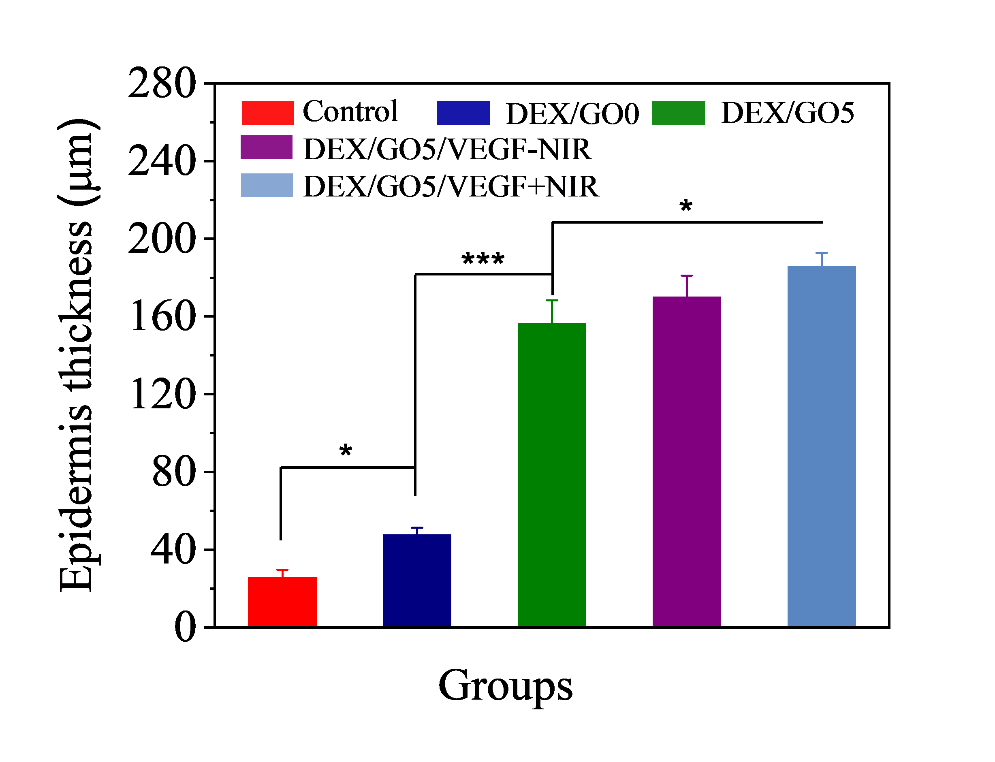


**Figure S8**. Quantitative width of epidermis thickness for each group.
